# Multi-sectoral action in non-communicable disease prevention policy development in five African countries

**DOI:** 10.1186/s12889-018-5826-6

**Published:** 2018-08-15

**Authors:** Pamela A. Juma, Clarisse Mapa-tassou, Shukri F. Mohamed, Beatrice L. Matanje Mwagomba, Catherine Ndinda, Mojisola Oluwasanu, Jean-Claude Mbanya, Misheck J. Nkhata, Gershim Asiki, Catherine Kyobutungi

**Affiliations:** 1 African Population and Health Research Center, Nairobi, Kenya; 2 Department of Public Health, Faculty of Medicine and Biomedical Sciences, University of Yaoundé I, Yaoundé, Cameroon; 3 Health of Population in Transition Research Group (HoPiT), Yaoundé, Cameroon; 4 Lighthouse Trust, Lilongwe, Malawi; 5 Human Science Research Council, Pretoria, South Africa; 6 Faculty of Public Health, University of Ibadan, Ibadan, Nigeria; 7 Anthropology Department, Catholic University of Malawi, Chiradzulu, Malawi; 8 Department of Anthropology, Durham University, Durham, England

**Keywords:** Non-communicable, Disease, Multi-sectoral, Policies, Africa

## Abstract

**Background:**

The rise of non-communicable diseases (NCDs) in Africa requires a multi-sectoral action (MSA) in their prevention and control. This study aimed to generate evidence on the extent of MSA application in NCD prevention policy development in five sub-Saharan African countries (Kenya, South Africa, Cameroon, Nigeria and Malawi) focusing on policies around the major NCD risk factors.

**Methods:**

The broader study applied a multiple case study design to capture rich descriptions of policy contents, processes and actors as well as contextual factors related to the policies around the major NCD risk factors at single- and multi-country levels. Data were collected through document reviews and key informant interviews with decision-makers and implementers in various sectors. Further consultations were conducted with NCD experts on MSA application in NCD prevention policies in the region. For this paper, we report on how MSA was applied in the policy process.

**Results:**

The findings revealed some degree of application of MSA in NCD prevention policy development in these countries. However, the level of sector engagement varies across different NCD policies, from passive participation to active engagement, and by country. There was higher engagement of sectors in developing tobacco policies across the countries, followed by alcohol policies. Multi-sectoral action for tobacco and to some extent, alcohol, was enabled through established structures at national levels including inter-ministerial and parliamentary committees. More often coordination was enabled through expert or technical working groups driven by the health sectors. The main barriers to multi-sectoral action included lack of awareness by various sectors about their potential contribution, weak political will, coordination complexity and inadequate resources.

**Conclusion:**

MSA is possible in NCD prevention policy development in African countries. However, the findings illustrate various challenges in bringing sectors together to develop policies to address the increasing NCD burden in the region. Stronger coordination mechanisms with clear guidelines for sector engagement are required for effective MSA in NCD prevention. Such a mechanisms should include approaches for capacity building and resource generation to enable multi-sectoral action in NCD policy formulation, implementation and monitoring of outcomes.

## Background

Multi-sectoral action (MSA) refers to actions that are undertaken by sectors outside the health sector—with or without the collaboration with the health sector—to attain health-related outcomes or influence health determinants [1]. MSA recognizes that the social and economic factors that influence a population’s health do not only lie inside the health sector but are also found within other sectors. MSA can help groups with disparate interests engender a more robust sense of institutional legitimacy and create a more unified front to address health priorities [2] such as the growing challenge of non-communicable diseases (NCDs) within Africa. In addition, MSA contributes to improvements in the health of the population by addressing social determinants of health. Social determinants of health create environments that influences population risk and individual behavior, leading to reduced inequities, healthier environments and increased coverage of health interventions at local, national, regional and global levels [3]. For instance, measures that can be taken outside the health sector towards preventing cardiovascular diseases include making healthy food more accessible by targeting agricultural production and the food industry. These activities can be implemented in concert with advocacy efforts for better nutrition and improved dietary habits [4].

In the recent past, MSA has been recognized as a priority in NCDs prevention and control [5]. The MSA emphasis was made during the first Global Ministerial Conference on Healthy Lifestyles and Non-communicable Disease Control held in Moscow in 2011 led to identification of four major areas for countries to address, including: strengthening government’s role in NCD prevention, developing multi-sectoral public policies and legal frameworks to reduce NCD risk factors, and strengthening health systems to respond to NCDs [6]. Furthermore, the World Health Organization (WHO) global action plan on NCDs (2013–2020) considers MSA as a major component of a ‘whole-of-government’ approach that involves various public sectors working in an integrated manner across their portfolio boundaries to achieve a shared goal [7]. Each sector in plays different roles. For instance increasing taxes and enforcing advertising bans or restrictions on tobacco and alcohol requires the involvement of sectors such as finance, health, and information ministries, the legislative arms of government, law enforcement, and the media [7]. Multi-sectoral action processes requires coordinated responses within and between sectors and creation of coordination bodies such as cross-ministerial executive committees, task forces, action teams and joint plans that outline shared inter-departmental goals with integrated budgets [8]. Involvement of different sectors also helps foster a sense of ownership among those designing and implementing different aspects of health programs [2]. Evidence on how MSA has worked in the context of NCD prevention in Africa is scarce.

This paper reports on findings from a broader multi-country study that was conducted to generate evidence on NCD prevention policies and the extent to which and how MSA was applied in developing the NCD prevention policies in five sub-Saharan African countries including Kenya, Malawi, Nigeria, Cameroon and South Africa. In this study we defined policies as written and unwritten authoritative government statements that are in place to guide national NCD prevention interventions. We focused on policies around the major NCD risk factors: tobacco control, control of harmful alcohol consumption, unhealthy diets and physical activity.

Walt and Gilson’s framework of policy analysis focuses on four elements including policy contents, actors, processes and context [9]. Policy content includes the policy objectives, the way the policy is designed, whether there is an accompanying implementation plan and specific mechanisms through which the policy should be actualized. The actors include those involved in the policy processes and their roles. The processes include the different stages of the policy-making process and the strategies employed to involve various actors. The context include the political climate and management structures; socio-cultural, economic or technological changes; and the development agendas of governments and development partners [9]. In this paper, we focus on the actors, in particular the government sectors and other stakeholders involved in the NCD prevention policy development processes. In addition, our analysis applied McQueen’s framework for inter-sectoral governance which focuses on the role of governance in tackling the social determinants of health through the emerging policy practice of “Health in All Policies” that is central to the envisaged NCD prevention efforts [10]. McQueen’s framework describes several inter-sectoral governance structures and their actions in promoting “Health in All Policies”. The governance structures include those at central government level, parliament level, civil service level, funding arrangements and mechanisms for engagement beyond government with other sectors. Depending on the policy, these governance structures may play different roles to initiate or facilitate a policy process and its implementation. These actions include evidence support, setting goals and targets, coordination, advocacy, monitoring and evaluation, policy guidance, financial support, providing legal mandates, and actual implementation and management [10].

## Methods

The broader study applied qualitative multiple-case study design [11] to capture the policy contents, contexts, processes and actors. Each of the five country teams analyzed multiple policies based on each risk factor, thus forming a single case and multiple case studies within each country report. The broader study was coordinated by the African Population and Health Research Center (APHRC) staff comprising of the principal investigator, a research manager, and a research officer. APHRC awarded fellowships to research teams from six countries to fund a mid/senior level research fellow, a PhD student, a research assistant, and an additional senior researcher to help manage the project in each country. The countries were selected to ensure geographical spread and based on the strengths of proposals submitted by the countries’ research teams from the countries. APHRC and the country team developed a toolkit to guide the study implementation. The tool kit included description of the study background, the objectives, and the procedures for data collection, ethical considerations, interviewing process, data management and analysis procedures.

### Data collection

In each country, data were collected through document reviews and key informant interviews with decision makers in various sectors. Each country team conducted document reviews, focusing on country-specific policy documents as well as policies from other low and middle-income countries. The documents were obtained from government ministry websites and offices, relevant donor or non-governmental organization and development partner websites. The documents reviewed included policy documents and statements, strategic plans, program plans, guidelines, protocols; local print media for references to policy changes, meeting minutes and journal articles. Data extracted from the documents included years in which relevant policy changes occurred and the events leading up to those decisions. Some key documents date back to the 1970s or 80s. An excel template was used to extract the information which was used during analysis.

Interview guides were informed by the Walt and Gilson framework of policy analysis [9]. Questions included the context in which the policy was developed, the policy content, actors involved in the process, and the implementation status, how MSA was employed or not, the processes undertaken to ensure MSA and barriers and facilitators in the process. During field worker training, each team piloted the guide and the interview guide was revised based on feedback from the pilots. Countries used the final interview guides with minor adjustments to fit their context if necessary. Interviews were conducted in English and the venue for the interview were mutually agreed upon by the research team and the participants. These venues were free from distractions and other security risks; and were conducted in a privacy. All interviews were digitally recorded, transcribed, cleaned, saved in word format by the country research teams with identification codes on password-protected servers.

### Data analysis

The research team developed an analysis plan during a qualitative data analysis workshop. A common codebook was developed based on the Walt and Gilson policy analysis framework elements. In each country, transcription of the interviews was done, and NVivo software was used to manage the data from qualitative interviews. Interview data were triangulated with document review data. For this paper, we focus on a synthesis of findings on actors and how MSA was applied across countries and across the four major NCD risk factors, from the five country study case studies.

## Results

Data collection began in 2014 and ended in 2016. Table 1 presents the summary of data sources. In total we reviewed 276 documents and interviewed 202 participants from all the five countries. Majority of the participants in all countries were from the health sector. This happened because majority of those who participated in policy processes and those who were willing to participate in the study were from the health sector. Despite the key informants being drawn from MOH, they provided relevant information pertaining to the involvement of other actors.

**Table 1:** Data sources

Source	Cameroon	Kenya	Malawi	Nigeria	South Africa	Total
Total Documents Reviewed	86	40	10	43	97	276
Interviews by sector						
Agriculture	0	2	4	0	1	7
Communications/Information	6	0	0	3	2	11
Economy and Planning	1	3	0	0	0	4
Education	23	1	0	2	2	28
Environment	1	0	0	0	0	1
Finance/Labor	2	2	1	3	1	9
Health	3	15	18	10	16	62
Industry/Trade	0	2	0	5	0	7
Justice	0	0	0	1	2	3
Law enforcement	0	1	2	0	2	5
Legislature	0	3	0	1	0	4
Trade and Industry	6	1	1	1	6	15
Academic and Research	0	4	2	12	8	26
Professional Associations	0	1	1	1	1	4
Other Ministries	1	0	3	3	3	10
Parastatals	0	4	0	2	0	6
Total	43	39	32	44	44	202

### NCD prevention policy status in the study countries

The countries have developed various NCD prevention policies targeting the major NCD risk factors. The policies range from legislation, comprehensive strategic and actions plans as well as government decrees. Table 2 provides an overview of current policies in each country. Details on the policy interventions and development processes including timelines are presented in another paper that is part of this supplement [12].

**Table 2:** NCD prevention policies in the countries

Policies	South Africa	Nigeria	Kenya	Cameroon	Malawi
Tobacco control legislation	√	√	√	√	X
Alcohol legislation	√	X	√	√	√
Food and nutrition security and policy	√	√	√	√	√
Nutrition action plan	√	√	√	√	√
Salt legislation	√	X	X	X	X
Physical activity	√	X	X	√	X
MOH NCD strategic plan/action plan	√	√	√	√	√

### Multi-sectoral engagement during NCD policy development processes

**Fig. 1:**
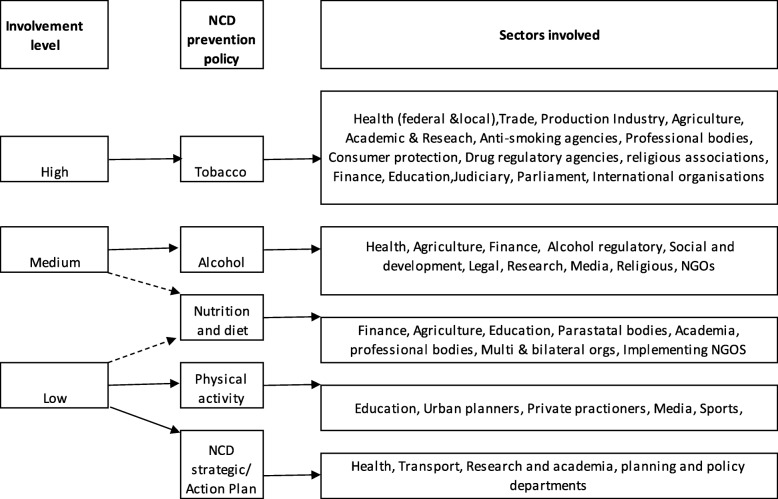
Sectors Involved in NCD prevention Policies

From the country case studies, different government sectors and departments, private sector, associations/boards, international organizations and local NGOs, have been engaged in NCD policy-making processes in the five countries. However, the number of relevant sectors involved in developing policies for individual risk factors varies. As shown in Fig. 1, involvement of other actors in policy development was high in tobacco, followed by alcohol, nutrition and diet and physical activity policies and with NCD strategic action/plan. From the country case studies, Malawi had more actors involved in the alcohol policy than all other countries followed by Cameroon, Kenya and South Africa [13]. Nigeria had the least number of actors for alcohol policy [14]. For nutrition and diet policy, only Nigeria and Malawi had some stakeholders involved, while the rest of the countries had low level of engagement of other actors.

From the country case studies, the health ministries were actively involved or led drafting of most of the policies. Engagement of different sectors varied from passive to more active involvement in policy formulation processes. Although most countries had at least nominal engagement of sectors outside the health sector during the development of NCD related policies, most of the countries indicated that this engagement was limited to providing information or viewpoints and was not a true collaborative effort. Most countries added other sectors to the conversations after the policies had been drafted, thus limiting their contributions. The country case studies reveal failure to engage some relevant sectors in the process like the finance sector which is one of the most important sector. In South Africa, participants were concerned that several institutions and entities involved in the sale or provision of food to the public were not involved in salt legislation. In almost all the countries, the development of the NCD Action Plan was led by the health ministry with engagement of health-related NGOs/CSOs and little involvement of other government sectors.

### Mechanisms for multi-sectoral actions

To describe the mechanism of engagement we apply the elements described in McQueen’s framework for inter-sectoral governance. The elements include governance structures that countries have put in place to guide MSA processes. We also describe the actions identified including evidence support, coordination, advocacy, financial support, providing legal mandates as well as implementation, monitoring and evaluation.

#### Governance structures and committees

From the country case studies several structures were established but they varied in countries and across policies for each risk factor as shown in Table 3. In addition they were not well established for long term engagement as many tended to be ad hoc technical working groups or taskforces. South Africa established both inter-ministerial and parliamentary committee under the direction of the Department of Social Development and the Department of Health, to drive all NCD related policies. Nigeria established an inter-ministerial National Tobacco Control Committee in 2016 to guide tobacco control policy and program development. Malawi established an inter-ministerial committee for drugs and alcohol control to oversee implementation of the alcohol policy at both central and local levels through sub-committees.

**Table 3:** Governance structures and committees

Governance Structures	Cameroon	Kenya	Nigeria	Malawi	South Africa
Inter-ministerial committees	For tobacco	–	Tobacco Legislation	For drugs and alcohol	All policies
Parliamentary committees	–	Tobacco legislation	None		All policies
Interdepartmental units	–	-NCD unit -Nutrition unit	NCD Division, Tobacco Unit Nutrition unit	-NCD Unit -Health Education Unit for healthy lifestyle promotion	–
Multi-sectoral expert/Technical working group/Taskforce	EWG for tobacco replaced in 2015 by the national multi-sectoral committee for tobacco control	–	NCD strategic plan/action plan Tobacco Legislation	Task Force for Alcohol Policy development National NCD sub-Technical Working Group	All NCD policies
Departmental Committees		Nutrition policies	Tobacco and Nutrition Policies	MOH Senior Management Committee	
Joint Budgeting	None	None	None	None	None
Delegated financing	None	None	None	None	None
Public engagement	–	–	Tobacco policy	Alcohol policy	All NCD policies
Stakeholder engagement	Done for some tobacco and alcohol policies	Tobacco and alcohol policies	-Tobacco and Nutrition Policies -NCD strategic plan/action plan	Alcohol policy	Done for all policies
Industry Engagement	Done during Tobacco and alcohol tax policy development process	Done to a small extent during the tobacco control policy process	Tobacco policy development	Done during Alcohol policy development process	All NCD policies

In most of the countries there were expert/technical working groups formed for various policies. In 2004, Cameroon created a multi-sectoral expert group on tobacco to examine evidence of smoking and its impact on public health and to facilitate the formulation of policy on health warnings for tobacco products. This group became inactive for many years till 2015 when a national multisectoral committee for tobacco control was created and is active to date.

However, the Cameroon government released several circulars and not comprehensive policy documents, thus experts were not engaged.

In the case of salt reduction in South Africa, the technical working group had representatives from different sectors that provided diverse perspectives about the content of the salt-reduction legislation. Malawi has a national NCD technical working group that functions under the Essential Health Package TWG as one of the sub-groups that steer interventions on NCDs their risk factors. However, membership has an over representation of the health sector since the working groups are instituted by the Ministry of Health. Malawi also formed an ad hoc multi-sectoral national task team for alcohol policy development led by the Ministry of Home Affairs and Internal Security in 2009. In 2011, the Ministry of Health took over the leadership and coordination role as alcohol control was placed under NCD control program. An example of a well-established permanent structure in Kenya is the National Authority for the Campaign against Alcohol and Drug Abuse that operates under the Ministry of Provincial Administration and Internal Security to coordinate other sectors and non-state actors in developing drug and alcohol policies. Kenya was in the process of establishing an inter-agency coordinating committee for NCDs that would involve all the relevant sectors in NCD policy development and prevention activities.

### Multi-sectoral engagement actions

#### Evidence support

In many instances, NGOs, research and academic institutions participated by providing country-specific data on the burden of NCDs and risk factors which served as tools for advocacy. Evidence included global scientific evidence that links tobacco smoking and alcohol consumption to risks such as cancers, cardiovascular diseases and chronic respiratory. Evidence was used to serve as a call to action for various actors who shared a common interest and need to develop tobacco and alcohol policies to reduce the rising negative health impacts. For tobacco, most countries initially relied on the evidence from the Global Youth Tobacco Survey, 2008 [15], and the Global Adult Tobacco Survey, 2012 [16], to guide the formulation of the Tobacco Act and the NCD Strategies. Additional evidence existed on the economics of tobacco including the costs and benefits of tobacco consumption. In South Africa, the salt reduction policy was preceded by many years of academic research that demonstrated the prevalence of high salt consumption in common foods and a willingness of major food producers to change their production protocols after research showed no impact on their sales.

#### Coordination

From the case studies, coordination happened across the existing departmental structures and non-governmental actors but with weak linkages at higher levels. Coordination activities involved communication and invitation of relevant sectors to attend planning meetings and draft or comment of the policy contents. Coordination at this level was enabled through technical/expert working groups and advisory committees formed to facilitate policy drafting in most of the countries. In Nigeria, the Ministry of Health inaugurated an advisory committee to coordinate implementation of the 2015 Tobacco Act in 2016. All the countries have set up NCD coordinating department/units within their health ministries to spearhead policy development by engaging other sectors.

#### Advocacy

In all the countries, NGOs and civil society conducted considerable advocacy that led to policy development and engagement of various actors. In South Africa, NGOs, such as Soul City, advocated for the regulation of alcohol exposure and support for the bill on banning alcohol advertising. International organizations such as the Campaign for Tobacco Free Kids provided technical support in Nigeria. In Kenya, various NGOs advocated for and supported tobacco and alcohol policy formulation. In Malawi, NGOs acted as the catalysts for initiating the policy formulation process and were the drivers of the process itself by coordinating stakeholder input, providing technical assistance and providing financial assistance. Coalitions and networks were also formed to sustain the implementation of some of the policies. These networks tend to engage various sectors in the process. The Nigeria Tobacco Control Coalition and Environmental Rights Action/Friends of the Earth Nigeria and others advocated for the passage of the Tobacco Control Act. Some professional associations also became part of advocacy and coalition networks. In South Africa, the National Campaign Against Smoking continues to lobby against tobacco smoking and also counsel smokers who would like to quit through managing a toll-free Quit line where smokers can call anytime and receive tobacco cessation support.

#### Financial support

From the case studies, countries reported that funds from the government was minimal and they were not quantified. NCD interventions had no budgetary allocation and most of the interventions were implemented within the health sector budget, which is low. Apart from South Africa, other countries reported that International organizations providing expertise and funding for meetings as well as for implementation of some of the interventions.

#### Providing legal mandates

Legal mandates were often implemented through the parliamentary committees and the technical committees where legal sector were involved. This was evident in formulation of tobacco and alcohol policies in Kenya and South Africa where policies drafted at sector levels go through the Department of Justice for drafting into a bill. The bill is then debated in cabinet and approved for release to the public for inputs. It is at the point of public consultation that multi-sectoral action takes place in policy-making and this happened in formulation of tobacco and alcohol policies in the two countries. The public whether as individuals or groups can provide submissions into a draft bill, attend public hearings on a particular bill and give their views whether in support or in opposition to the bill. All NCD policies in South Africa went through the same process [17].

#### Implementation, monitoring and evaluation

In most of the countries, the policy documents propose involvement of multiple sectors at the level of implementation but the extent of this engagement in actual implementation is not evident in the data. Multi-sectoral engagement seemed to be higher at policy development stage than implementation stage. In South Africa, for instance, multi-sectoral action in the implementation of NCD policies occurred when implementing the ban on tobacco smoking in public places. The public, employers and local government played an active role in enforcing the ban on smoking. In terms of implementing programmes targeting physical inactivity, multi-sectoral action has been evident in the collaboration of the national department of health with the department of Sports and recreation as well as the department of social development. In such collaborations, the private sector has also been involved in sponsoring specific activities such as games for the elderly. The business community is also involved in implementing activities targeting physical activities particularly in the convening of charity walks/ runs to raise awareness and funds for prevention activities such as breast cancer. NGOs also work with national departments to support various prevention activities. Evidence of engaging sectors in monitoring and evaluation was not found in all countries.

### Challenges to multi-sectoral action

These case studies revealed several cross cutting challenges to MSA.

#### Differences in interests, priorities and objectives

In some instances, participation was hindered by the differences in interests and objectives of the different sectors. In South Africa, the departments of Social Development, and Health and the police are concerned about the negative health impacts of alcohol, while the departments of Finance as well as Trade and Industry are concerned about the loss of revenue from taxing alcohol consumers and also about job losses in the alcohol and advertising industries. So far there has been no consensus between the different state departments. Strong opposition from the media and sporting fraternity seems to have ‘killed’ the bill that proposed banning alcohol advertising before it was presented to the public for comment. For some countries like in Malawi, this is because government is caught in a dilemma of balancing between controlling tobacco consumption and its financial needs as tobacco is a major foreign exchange resource. From the findings, some decision-makers would not endorse policies related to NCD prevention because of their personal interests in the industries or the country’s economic interests. This was especially common in the case of alcohol and tobacco policies. In Nigeria, the differences in the interests and objectives of the different sectors were also observed.

Other major actors with different interests were unhealthy commodity industry. Industry interference led to delayed endorsement of alcohol (e.g., in Malawi) and tobacco (e.g., in South Africa) policies and has continued to complicate full implementation of policies due to legal challenges (e.g., tobacco in Kenya) (Juma et al. 2018; In this issue). Contrary to WHO recommendation, some countries involved the industries in the policy development processes. For instance, the Standard Organization of Nigeria, which regulates the production of manufactured goods and products, called for the involvement of the tobacco industry in the policy formulation, and subsequently involved the tobacco industry in the development of the 2014 Standard for Tobacco and Tobacco Products-Specifications for Cigarettes.

#### Lack of adequate resources

Most of the countries reported insufficient financial resources allocated to developing NCD prevention policies and for engaging multiple sectors in policy implementation activities. Inadequate finances and human resource capacity meant that policies were not being implemented. There seems to have been an over-reliance on NGOs to support certain aspects of policy formulation and implementation. However, NGOs do not have sufficient funding to sustain their activism for NCDs. In many instances, NGOs have to fundraise for their activities and this limits the amount of financial resources and time that can be committed to educating the public and creating awareness around the NCD risk factors. Although funding for treatment for NCDs was available in public sector health facilities, the lack of sufficient resources meant that more often there was little that could be done to increase awareness regarding NCD prevention and control.

#### Lack of political will

Inadequate political will hindered both policy formulation and MSA. While all countries endorsed the Political Declaration from the United Nations General Assembly in 2011, there had been little commensurate action on the ground in allocating the needed resources to operationalize these commitments with the involvement of multiple sectors. In most of the countries, governments were slow in taking action and often lacking the political will to formulate policies to address the NCD risk factors. In Malawi, this is also shown by failure to ratify the WHO FCTC. In Cameroon, even if the FCTC was ratified in 2006, government granted huge subsidies to tobacco farmers in the country.

#### Coordination challenges

From case studies, many countries had no clear rules or coordination frameworks to guide working with other sector. In many instances, there was no guarantee that targeted stakeholders would participate or that that all relevant stakeholders were included, as participation was not compulsory but rather voluntary. Kenya, Malawi, and Nigeria reported complexities in sector operations and high staff turnover that made it difficult to have the same individuals participating consistently and maintaining similar views. In many instances, some sectors were represented by different people at different meetings and workshops. In some cases, there was no proper handover to create continuity in subsequent meetings. This was most common in government ministries and departments. In the case of South Africa, coordinating a large volume of different people was the most prominent challenge coupled with resource challenges. In Malawi and Kenya, coordination was difficult because sectors had very different views and it was challenging to synthesize those diverse views into a single coherent plan.

#### Lack of awareness by the relevant sectors

There was lack of awareness about NCDs and their risk factors among the population in the countries. In countries such as Kenya, Cameroon, Malawi and Nigeria, NCDs had not been given priority in the past as compared to other diseases (communicable), and so awareness among non-health sectors was even lower. Many sectors other than the health sector were unaware of their potential contributions to NCD prevention. This challenges implicit assumptions about the levels of awareness among players in all sectors of their role in health generation and maintenance. NCD prevention was assumed to be a health sector issue and so that sector spearheaded policy development to address these risk factors.

#### Competition among government sectors

Another barrier was competition among sectors, particularly around leadership of some policies. In Malawi, for instance, during the development of alcohol policy there was conflict between the Ministry of Health and the Ministry of Trade and Industry about who would lead the process. This competition also affected implementation. In Malawi, there was a perception that each sector has its own mandate and that it didn’t necessarily have to implement the policies/guidelines coordinated by other sectors. In Nigeria during the formulation of the Tobacco Act, there was competition between the Federal Ministry of Health and the regulatory organizations over which was the most appropriate ministry to lead the tobacco control policy process. In South Africa, the priorities of the Department of Trade and Industry, and Treasury clashed with the public health concerns of the Department of Health and Social Development. As a result, passing a bill to ban alcohol advertising became complicated and fraught and was eventually withdrawn.

## Discussion

Findings from the case studies show increased recognition of NCDs as a major problem that requires engagement of different actors and sectors in prevention interventions. In this study, multi-sectoral engagement in NCD prevention policy process varied by each risk factor and it happened more at the policy formulation stage and less during implementation. Application of McQueen’s framework for inter-sectoral governance has enabled us to describe the governance structures and actions that enabled multi-sectoral action in NCD prevention policies from the five country case studies.

The presence of national multi-sectoral governance and coordination structures or mechanism to oversee NCD policy engagement beyond the health sector has facilitated MSA in NCD policy development in some countries. Such structures are useful for coordination and have been promoted as essential elements for effective MSA [18]. However, it emerged that such mechanisms had not been well established in most of the study countries, thus ad-hoc expert/technical working groups were used to facilitate policy development. Furthermore, coordination of the different sectors to work together in policy development was made difficult due to lack of clear guidelines for engagement with other sectors. Lack of such guidelines could have been the reason low levels of sustained engagement with other sectors. Other East African countries such as Uganda and Rwanda have recently created NCD multi-sectoral coordination structures but they are yet to engage on a regular basis [19]. Furthermore coordination of actors for sustained action has been difficult in the study countries.

Although countries included multiple actors in NCD prevention policy processes, active engagement of relevant government sectors is still inadequate at both the formulation and implementation stages. The health sectors in all countries tended to take lead in designing and implementing the policies and in initiating collaborative efforts with other sectors after policies are drafted. Thus, majority of the actors were from the health sector, either from the ministries of health as well as private sector or NGOs implementing health activities. While the health ministry’s leadership would seem to be a natural fit for addressing NCDs it is well recognized that MSA requires involvement of non-health sector at all stages of the policy process. The health sector should display a more collaborative and distributive leadership to enable effective governance and build leadership capacity across sectors and all levels of government to cultivate champions in different sectors who can agree on common objectives [18].

Actions such as evidence sharing and advocacy were the main driving factors to multi-sectoral engagement. Some actors availed scientific evidence showing the negative impacts of the risk factors on the population, particularly for tobacco smoking and alcohol consumption. The initial evidence cited by most of the countries seemed to have emerged globally; however, countries have started generating their own contextual evidence to drive implementation of the “best buys” interventions within the context of MSA. Locally generated evidence could have shaped the policy goals and strategies but also acted as a driver for other sectors to engage in the NCD prevention policy processes. This evidence was also used to drive advocacy activities that brought together different groups of stakeholders. These groups of stakeholder tend to form coalitions and networks to sustain policy formulation implementation as well as to engage various sectors in the process and will be of importance in driving future implementation.

A common barrier to MSA was conflict of interest, especially by the political leadership in the countries particularly with regard to tobacco and alcohol policies. Such conflicts may slow or halt both policy development and engagement of different sectors. One major negative effect of actor interest was shown through industry interference with the policy development and implementation process. In particular tobacco industry interference with policy process have been evidence in almost every country in LMIC [18]. Governments tend to allow industries to contribute to policy processes despite the fact industries will want to serve their interest in making profits rather than addressing public health impact of their products.

Another key barrier was sectors’ lack of awareness of their potential contribution to addressing NCDs. The findings challenge implicit assumptions about the levels of awareness among players in all sectors of their role in health generation and maintenance. One reason for this low awareness may be the assumption that NCD prevention is a health sector issue, and this sector tends to regard NCD issues as its domain and, thus, spearheads corresponding policy development. Countries that engage in MSA may take longer to enact policies because of the time required to ensure that all stakeholders share an understanding of the problems and the solutions; this understanding may ultimately make the policies more effective.

Inadequate political will was identified as a barrier to both policy formulation and MSA in most of the countries. This is reflected in the inadequate government resource allocation for NCD prevention in general and for multi-sectoral engagement in policy development and implementation. Inadequate financial allocation for NCD prevention has been an issue of concern in developing countries where domestic health financing is dominated by out of pocket payments that are only used for NCD care in health facilities [20]. Also of note was inadequate resources in terms of technical capacity and sustained funding for MSA processes in policy development, implementation and review of outcome. To address such challenges some, countries like Uganda are implementing initiatives including multi-sector partnerships focused on capacity building and health systems strengthening for NCD prevention; a model civil society collaboration leading a regional coalition and a novel alliance of parliamentarians lobbying for NCD policy [21].

## Conclusions

This study shows that countries are making efforts to incorporate multi-sectoral action in policies to address NCDs. However, various challenges and gaps still exist in multi-sectoral action in NCD prevention policy development and implementation that need to be addressed in order to improve the health status of each country’s citizens. The following are the recommendations:

1. There is need to strengthen governance and coordination structures across sectors and by levels to ensure that all relevant sectors are engaged in NCD prevention initiatives. Governments should *engage* all the relevant stakeholders to develop clear guidelines on how different sectors should work together to develop NCD prevention policies and programs, especially in relation to the WHO “best buys” interventions. This strategy requires a mapping of the different actors and stakeholders, an assessment of their awareness on NCDs and MSA and an investigation of platforms that can be effectively used to reach them. The strategy would also entail sequencing of the actions with quick-wins implemented in the strategy’s early stages and the more complex actions being undertaken later, as countries gain more experience in MSA operationalization. Further guidelines to guide the countries in sustaining MSA structures are provided in the WHO framework for multi-sectorial steering committees [22].
2. Countries need to enhance NCD *awareness* among various sectors and address issues of conflict through employing a strong advocacy and communications strategy on MSA for NCD prevention. An effective strategy requires identification of: i) Whom to target with messages; ii) What to communicate in messages; and, iii) How to communicate the messages.
3. The health sector should enhance its MSA *leadership* role by creating awareness and bringing other sectors on board. The health sector should be more engaged in relevant activities of the other sectors that relate to NCD prevention, such as monitoring tax reforms and budgets.
4. These strategies should *incorporate existing mechanisms* to operationalize the global agenda on NCDs at country levels and within the context of sustainable development goals.
5. Sustainable joint financing mechanisms need to be established for effective implementation of MSA for NCDs prevention. Together, these strategies can create a more effective infrastructure of stakeholders to establish and sustain effective MSA in formulating and implementing the NCD policies in the African countries.
6. Implement strategies to counteract industry interference that blocks the implementation of NCD prevention measures. With regard to tobacco industry interference, several measures have been proposed, including reduction of participation of tobacco industry in policy development and improvement in transparency in dealing with the industry [23].

## Abbreviations

ANPPA: Analysis of Non-communicable Disease Prevention Policies in Africa
CSO: Civil Society Organisation
FCTC: Framework convention on tobacco control
MOH: Ministry of Health
MSA: Multi-sectoral action
NCD: Non-communicable disease
NGO: Non-governmental organisation
WHO: World Health Organization
